# Lung cancer with partial anomalous pulmonary venous connection in a different lobe: a case report

**DOI:** 10.1186/s13019-021-01505-y

**Published:** 2021-05-01

**Authors:** Hikaru Watanabe, Naoki Kanauchi, Kouhei Abe, Soumei Matsuo

**Affiliations:** Department of General Thoracic Surgery, Nihonkai General Hospital, 30 Akiho-cho, Sakata, Yamagata, Japan

**Keywords:** Partial anomalous pulmonary venous connection • lung cancer • pulmonary to systemic flow ratio

## Abstract

**Background:**

Anomalous pulmonary venous connection (APVC) is a congenital malformation in which the pulmonary veins connect to the systemic venous system but not to the left atrium. APVC can be classified as total or partial (PAPVC). PAPVC is rare among surgical patients with lung cancer, and most cases are detected incidentally during surgery. We herein report a patient with lung cancer in whom PAPVC was diagnosed before surgery, which made it difficult to determine the surgical procedure.

**Case presentation:**

A 71-year-old man was followed-up as an outpatient after surgery for renal cell carcinoma. Chest computed tomography showed a 22-mm nodule in the right lower lobe and PAPVC in the right upper lobe. He was diagnosed with lung adenocarcinoma (cT1cN0M0 stage IA3) and scheduled for surgery. Preoperative catheterization showed a pulmonary to systemic flow ratio (Qp/Qs) of 1.64 and mean pulmonary artery pressure (MPAP) of 16 mmHg. Surgical repair of PAPVC is indicated when a patient is symptomatic and has a Qp/Qs ≥1.5–2.0. The patient was scheduled for right lower lobectomy, but postoperative worsening of right heart strain was considered. Concomitant PAPVC repair was therefore considered, but he had no atrial septal defect and was asymptomatic; therefore, PAPVC treatment was considered unnecessary. However, we planned to perform concomitant PAPVC repair if his circulatory dynamics worsened during surgery or if his MPAP exceeded 25 mmHg. His MPAP was 20 mmHg and his circulatory dynamics remained stable, and right lower lobectomy was therefore completed. His postoperative course was favorable. Follow-up catheterization at 6 months showed a Qp/Qs of 1.19 and MPAP of 18 mmHg, with no evidence of increased right heart strain. There was no evidence of right heart failure or recurrence of lung cancer at last follow-up at 18 months after surgery.

**Conclusions:**

We present a case of right lower lung cancer complicated by PAPVC in the right upper lobe. This case suggests that concomitant repair of PAPVC in the right upper lobe may not be necessary when performing right lower lobectomy, although the patient’s Qp/Qs and MPAP should be considered.

## Background

Early-stage non-small cell lung cancer should be treated surgically whenever possible. Although some patients also have minor congenital anomalies, such as pulmonary arterial, venous, or bronchial variations, most of these do not cause serious problems during or after lung resection. However, the presence of a vascular shunt in another lobe of the lung may cause fatal problems in patients undergoing major lung resection. Partial anomalous pulmonary venous connection (PAPVC) is a rare congenital anomaly in which the pulmonary venous system drains into the systemic circulation instead of into the left atrium. If the lung cancer is located in the same lobe as the PAPVC, lobectomy allows a radical cure for both conditions; however, if the PAPVC is located in a different lobe from the lung cancer, major lung resection is required, which could increase the shunt flow volume and lead to right-sided heart failure. The treatment plan for patients with PAPVC requiring resection of a different lung lobe from that affected by lung cancer thus requires careful consideration. Herein, we report the case of a 71-year-old man with lung cancer in the right lower lobe and a PAPVC of the right superior pulmonary vein with the azygos vein.

## Case presentation

**Fig. 1:**
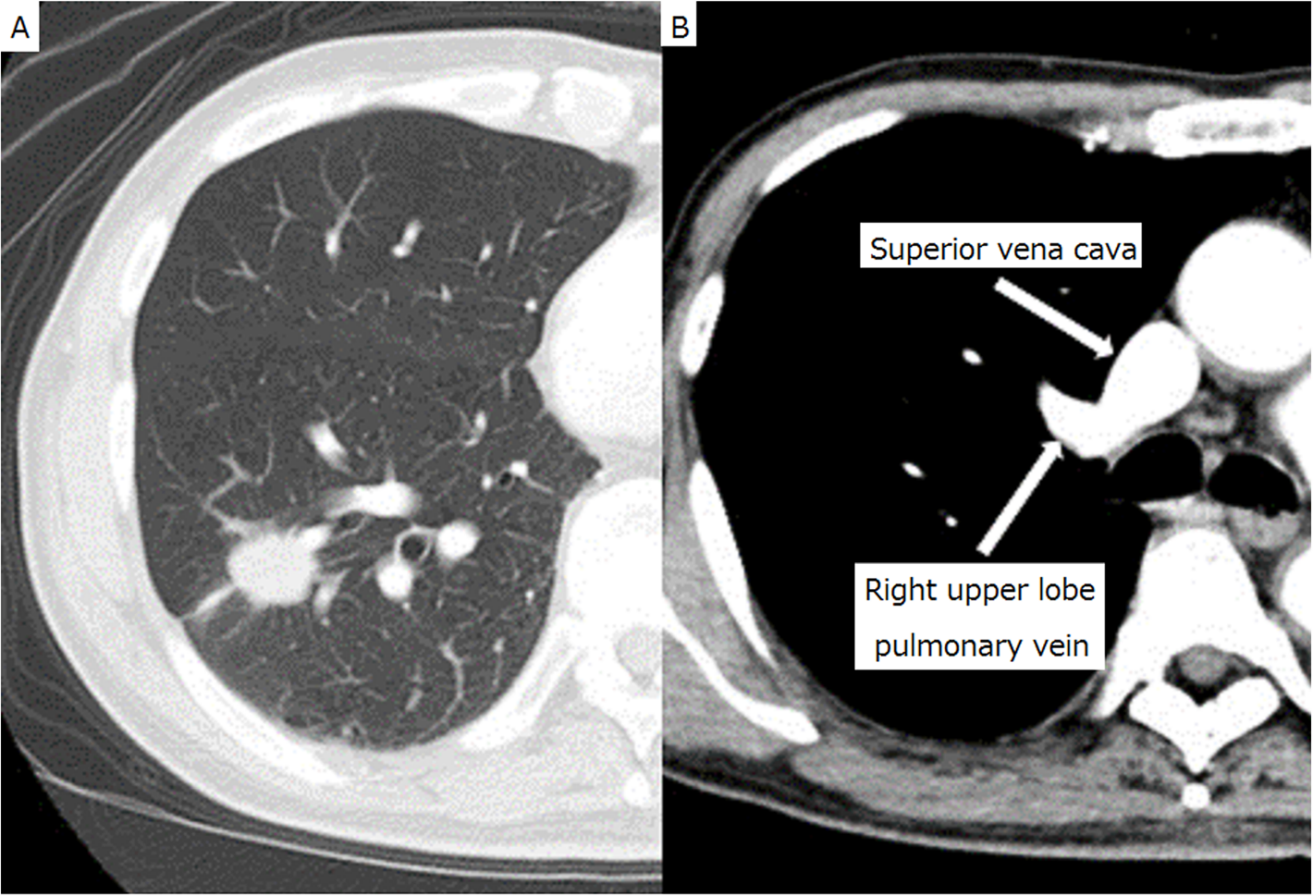
Computed tomography. Thoracic computed tomography showed a tumor shadow in the right lower lobe (**a**) and an enhanced anomalous vein (**b**)

An asymptomatic 71-year-old man presented with an abnormal lung nodule detected by computed tomography (CT) during a regular postoperative checkup for renal cancer. CT revealed a nodule in the right lower lobe and a PAPVC of the right superior pulmonary vein with the azygos vein (Fig. 1). Transbronchial biopsy revealed adenocarcinoma. The echocardiogram and electrocardiogram showed normal cardiovascular activity without atrial septal defect. Although the patient was asymptomatic, cardiac catheterization revealed a pulmonary to systemic flow ratio (Qp/Qs) of 1.64, and his mean pulmonary arterial pressure (MPAP) was 16 mmHg (27/6 mmHg). Blood gas analysis revealed age-normal partial oxygen (79.7 mmHg) and carbon dioxide pressures (41.5 mmHg). Pulmonary function tests showed forced expiratory volume in the first second of 2.29 L (78% predicted) and forced vital capacity of 3.34 L (93% predicted). His lung cancer was considered to be resectable clinical T1c N0 M0 stage IA3 and right lower lobectomy was planned; however, it was considered that this could cause fatal right-sided heart failure unless the PAPVC was repaired. The possible surgical approaches of right lower lobectomy for the lung cancer and concurrent PAPVC repair were discussed preoperatively with a cardiovascular surgeon.

We were concerned that the patient might be at risk of fatal postoperative right-sided heart failure without PAPVC repair. However, he had no atrial septal defect and was asymptomatic, and we therefore concluded that it was not necessary to treat the PVPAC. Based on these considerations, we decided to only perform concomitant repair of the PAPVC if his circulatory dynamics could not be maintained during surgery or if his MPAP exceeded 25 mmHg.

**Fig. 2:**
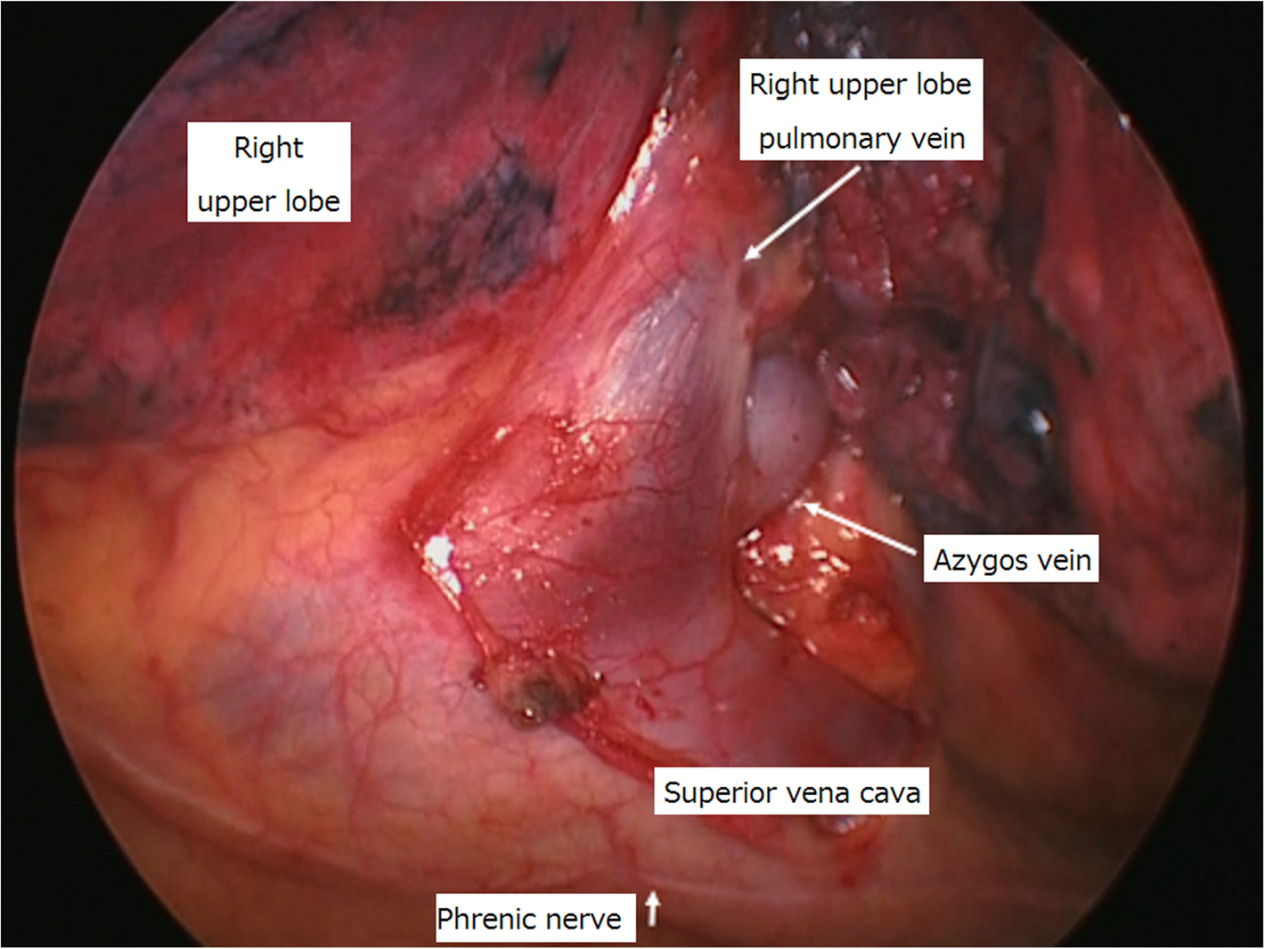
Surgical findings. The anomalous pulmonary vein was observed to drain into the azygos vein from the right upper lobe

The patient’s pulmonary artery pressure was carefully monitored during surgery using a Swan-Ganz catheter. His MPAP was 20 mmHg; therefore, we performed thoracoscopic right lower lobectomy, without repairing the PAPVC. The anomalous pulmonary vein was found on the anterior aspect of the right hilum. In accordance with the CT observations, we found a PAPVC of the right superior pulmonary vein with the azygos vein (Fig. 2). The patient had an uneventful postoperative course. The lung cancer was confirmed as pathological stage IA3 (T1c N0 M0). Postoperative cardiac catheterization 6 months after surgery revealed a Qp/Qs of 1.19 and MPAP of 18 mmHg (31/7 mmHg). He remained well with no relapse of lung cancer at 18 months after surgery.

## Discussion

PAPVC is a relatively rare congenital anomaly, with a reported incidence of 0.4–0.7% in the general population at autopsy [1, 2]. In children, PAPVCs are commonly associated with atrial septal defects and are located primarily on the right side. Conversely, a recent review of 29 adult cases of PVPAC found incidentally by CT showed that most were located in the left upper lobe (79%) and were very rarely complicated with atrial septal defects (4%) [3]. The surgical indication for the repair of symptomatic PAPVC repair is commonly a Qp/Qs > 1.5 [4, 5]. However, PAPVC without an atrial septal defect is generally asymptomatic and may be clinically insignificant. Nevertheless, surgical treatment is recommended for PAPCVs in patients with a Qp/Qs > 2.0, regardless of associated cardiac defects [6, 7].

Among 22 previous case reports of PAPVC associated with lung cancer, both conditions were located in the same lobe in 13 cases and in different lobes in 9 cases. Of these nine cases of PAPVC and lung cancer in different lobes, five patients did not undergo treatment for the PAPVC during pulmonary resection [8, 9]. The major lung resection required in such patients could increase the shunt flow volume and lead to right-sided heart failure. Black et al. reported a patient who died of severe right-sided heart failure after right pneumonectomy because the PAPVC involved drainage of the left superior pulmonary vein into the left brachiocephalic vein [10]. It is therefore necessary to carefully check the drainage of the contralateral pulmonary veins before performing pneumonectomy. Sakurai et al. reported a case of right PAPVC repair before left pneumonectomy for lung cancer [11]. PAPVCs are generally located on the right side, and cardiopulmonary bypass is required for their repair because of the shortness of the anomalous vein traveling to the superior vena cava (SVC) or right atrium. However, left-sided PAPVCs can be corrected by a simple repair, such as an end-to-side anastomosis directly to the left auricular appendage or the left atrium, or end-to-end anastomosis to the stump of the resected normal pulmonary vein. Extracorporeal circulation is not required for the operative procedure in these cases [12]. The treatment strategy thus depends on the location of the PAPVC.

In the current patient, the PAPVC was right-sided and its repair would require extracorporeal circulation and a Swan-Ganz catheter. However, because he had no preoperative symptoms of PAPVC, no atrial septal defect, and a Qp/Qs of 1.64 during preoperative cardiac catheterization, we decided not to perform concomitant repair of the PAPVC. However, we were prepared to perform a combined PAPVC repair under extracorporeal circulation if his circulatory dynamics could not be maintained during surgery or if his MPAP exceeded 25 mmHg. Furthermore, because of the possibility that the patient might develop right-sided heart failure over the course of several months after surgery, we did not perform mediastinal lymph node dissection in case the need for PAPVC repair arose postoperatively.

If lobectomy is performed in a lung without PAPVC, it would be difficult to predict the specific postoperative Qp/Qs values. Kurihara et al. reported the prediction of Qp/Qs after left lower lobectomy by performing balloon test occlusion in the left inferior pulmonary vein with cardiac catheterization [9]. However, the pulmonary blood flow on the pneumonectomy side may decrease over the course of several months after surgery, and the predicted Qp/Qs value may thus change. Moreover, the Qp/Qs in the present case was expected to increase postoperatively, but it actually decreased from 1.64 to 1.19. These findings suggest that patients with lung cancer and PAPVC in different lobes on the same side should first undergo lobectomy for the lung cancer, and should then undergo careful follow-up. Previous studies indicated that the need to repair a PAPVC associated with pulmonary resection depended not only on hemodynamic factors such as Qp/Qs, but also on the presence of an atrial septal defect and on surgical factors including the site of the PAPVC relative to the pulmonary resection and the extent of the pulmonary resection relative to the PAPVC [8]. Treatment reports from more similar cases are required to confirm our findings.

## Conclusions

We present a patient with PAPVC in the right upper lobe who required surgery for right lower lung cancer. This case suggests that concomitant repair of PAPVC in the right upper lobe may not be necessary when performing right lower lobectomy, but the patient’s Qp/Qs ratio and MPAP should be considered.

## Data Availability

Not applicable.

## Abbreviations

APVC: Anomalous pulmonary venous connection
PAPVC: Partial anomalous pulmonary venous connection
Qp/Qs: Pulmonary to systemic flow ratio
MPAP: Mean pulmonary artery pressure
CT: Computed tomography
SVC: Superior vena cava
